# Ezrin expression in circulating tumor cells is a predictor of prostate cancer metastasis

**DOI:** 10.1080/21655979.2021.2014710

**Published:** 2022-02-12

**Authors:** Zheng Chen, Jue Wang, Yangbai Lu, Caiyong Lai, Lijun Qu, Yumin Zhuo

**Affiliations:** a Department of Urology, The First Affiliated Hospital of Jinan University, Guangzhou, Guangdong Province, China; b Department of Pathology, The FirstAffiliated Hospital of Sun Yet-Sen University, Guangzhou, China; c Departments of Urology, Zhongshan City People’s Hospital, Zhongshan, China

**Keywords:** Prostate cancer (PCa), circulating tumor cells (CTCs), Ezrin, tumor metastasis

## Abstract

Metastatic prostate cancer (PCa) remains incurable and fatal. Previous studies have proven that circulating tumor cells (CTCs) and Ezrin are involved in PCa progression, metastasis, diagnosis, and prognosis. Therefore, we aimed to investigate the roles of CTCs and Ezrin in PCa metastasis. The expression of Ezrin was measured by qRT–PCR and immunohistochemical staining. The migration and invasion of PCa cells were evaluated. Additionally, clinical data from PCa patients were collected to analyze the potential roles of Ezrin expression in CTCs of PCa. The results showed that Ezrin expression was significantly upregulated in PCa tissues and 22RV1 and PC-3 cell samples. The overexpression of Ezrin promoted the migratory and invasive abilities of 22RV1 and PC-3 cells. Finally, the clinical data revealed that the expression of Ezrin in CTCs of PCa patients was significantly upregulated with the metastatic degree. Furthermore, after radical prostatectomy, CTCs from Ezrin-positive PCa patients were susceptible to tumor metastasis. Therefore, these results indicated that Ezrin expression in CTCs may offer novel insights into the prognosis and management of PCa.

## 1. Introduction

Prostate cancer (PCa) is one of the most commonly diagnosed tumors in males and the second leading cause of death in elderly men around the world [1,2]. In recent decades, great efforts have been made in the systemic and individualized treatment of PCa, but the incidence of PCa remains markedly upregulated in China [3]. A family history of PCa, increasing age, and ethnicity are some of the key risk factors for the development of PCa. However, therapeutic modalities for PCa include hormone therapy, radiotherapy or surgical removal, which are effective for the early stage of PCa but not for the advanced stage of PCa [4,5]. Moreover, tumor metastasis has always been a major obstacle to the treatment of PCa during the advanced stage. Lymph nodes and bones are the most common sites for PCa metastasis, which severely threatens quality of life and even survival time in PCa patients [6,7]. Therefore, further investigation of the potential mechanisms that contribute to the metastasis of PCa is of utmost importance, as these investigations might facilitate the identification of novel targets and the diagnosis of PCa metastasis.

Current diagnostic approaches for PCa patients rely mainly on the measurement of prostate-specific antigen (PSA) levels; 0.5 ~ 2 ng/ml is the normal range, and above 4.0 ng/ml is the abnormal range [8,9]. However, the US Preventive Services Task Force (USPSTF) recommends against any routine PSA-based screening for PCa due to its low accuracy [10,11]. Thus, developing a simple, accurate and novel marker that can better predict PCa disease and identify PCa metastasis is critically needed.

Circulating tumor cells (CTCs) are shed primary cancer cells that invade the bloodstream, enhance the growth and colonization of PCa cells in distant organs, and ultimately forme new metastatic lesions. It was reported that CTCs were candidates for predicting disease progression and survival or therapeutic effects in patients with PCa [12–14]. Hence, CTCs have gained increasing interest in tumor metastasis-related studies of PCa [15].

Ezrin, also called cytovillin or villin2, is a component of cell-surface structures and acts as a membrane organizer and linker between the plasma membrane and the cytoskeleton, belonging to the Ezrin, radixin and moesin (ERM) protein family [16,17]. Many studies have proven that Ezrin participates in the development of human cancer. For example, Elliott et al. proved that Ezrin was necessary for breast cancer metastasis [18], Elzagheid et al. verified that Ezrin expression was involved in poor survival in colorectal cancer [19], and Xu et al. revealed that Ezrin could be a prognostic factor and a predictor of potential lung metastasis in osteosarcoma [20,21]. Hence, we speculated whether CTCs and Ezrin, which are both closely related to tumor metastasis, are collectively responsible for predicting the metastatic process of PCa. To demonstrate this hypothesis, this article aimed to explain the expression levels of Ezrin in PCa, the role of Ezrin in the mobility of PCa *in vitro* and the indicative function of Ezrin expression in CTCs in the progression of PCa.

## 2. Materials and methods

### 2.1. Patients and specimens

Eighty PCa patients who provided written informed consent and approval were enrolled in this study in the Department of Urology, First Affiliated Hospital of Jinan University from January 2017 to December 2017. The inclusion criteria included patients with full clinicopathological information (tumor node metastasis, TNM stage, PSA level, Gleason score) and patients without surgical resection or other treatment before tissue collection (radio/chemo/hormonal therapy). The tissues were collected from biopsy. The detailed information is recorded in Table 1. We also enrolled 53 PCa patients who received radical prostatectomy. Among the 53 PCa patients, patients with positive/negative expression of Ezrin were compared after their surgeries. The clinical tissues and serum samples of these patients were collected and immediately stored in liquid nitrogen for research. This study was designed according to the ethical guidelines of the Declaration of Helsinki, and the Ethics Committee of Jinan University approved all protocols with clinical samples.

**Table 1.** Clinical information about 80 PCa patients

	Total	Localized PCa	PCa with lymph node metastasis	PCa with bone metastasis
Number of cases (N)	80	34	14	32
Age (Years)	66 (46 ~ 86)	66 (46 ~ 82)	66 (52 ~ 86)	66 (53 ~ 85)
PSA level	24.2 (0.9 ~ 2875)	24.2 (0.9 ~ 250.5)	15.5 (1.5 ~ 91.9)	58 (9.7 ~ 2875)
Gleason score (GS)	7.5 ± 1.5	7.1 ± 1.5	7.5 ± 1.3	8.1 ± 1.1
Mean of CTCs	7	3.2	6.3*	11**
Ezrin+ in CTCs	44 (55.0%)	2 (5.88%)	12* (85.72%)	30** (93.75%)

*indicates *p* < 0.05 vs localized PCa; **indicates *p* < 0.01 vs localized PCa.

### 2.2. Immunohistochemistry analysis (IHC)

The collected tumor tissues were fixed in 10% FA and embedded in paraffin. Then, the paraffin sections were cut into 4-μm sections and deparaffinized in xylene and descending alcohol concentrations. Citrate buffer (pH 6.0) was used for antigen retrieval at 100°C for 15 min to expose antigenic sites, and then the sections were returned to room temperature and washed with buffer solution for 5 min. After blocking with 5% BSA at 37°C for 30 min, the sections were incubated with primary antibody at 4°C overnight in a humidified chamber (Ezrin 1:200 dilution; Abcam, USA), followed by HRP-conjugated secondary antibody incubation for 30 min. Finally, coloration was performed with diaminobenzidine (DAB; Beyotime, China). The staining results were evaluated by 3 pathologists who were blinded to patient outcomes.

### 2.3. qRT–PCR examination

Total RNA from cell and tissue samples was extracted using TRIzol reagent (TIANGEN, China), and the concentration of total RNA was determined in a NanoDrop1000 (Thermo Fisher Scientific, USA). RNA was reverse transcribed using a PrimeScript™ II 1st Strand cDNA Synthesis Kit (TAKARA, Japan). Then, qPCR was carried out in an Applied Biosystems PRISM® 7500. All primers were synthesized by Sangon Biotechnology: Ezrin forward primer: 5ʹ-AGCTGTGAAGAGACTCTGTTTG-3ʹ and reverse primer: 5ʹ-CTTAGCTGTGAAGGAGAAAGC-3ʹ; 18S rRNA forward primer: 5ʹ-CCTGGATACCGCAGCTAGGA-3ʹ and reverse primer: 5ʹ-GCGGCGCAATACGAATGCCCC-3ʹ. The PCR conditions were as follows: 95°C for 30 s, then 40 cycles of 95°C for 5 s and 62°C for 30 s. The melting curve was recorded and used for data collection. After normalizing to the expression of 18S rRNA, the relative RNA expression was calculated using the 2^−ΔΔCt^ method.

### 2.4. Cell culture and transfection

The human prostate cancer cell lines 22RV1 and PC-3 were cultured in RPMI‑1640 medium (Corning, USA) containing 10% fetal bovine serum (FBS; Life Technologies, USA) supplemented with 100 units/ml streptomycin and penicillin at 37°C in a 5% CO_2_ atmosphere. Cells were subcultured using 0.25% trypsin/EDTA solution (Invitrogen, USA) every 2 ~ 3 days.

22RV1 and PC-3 cells were added to 6-well plates and incubated for 24 h at 37°C at 5 × 10^5^ cells/well. Then, cells were transfected with blank, negative control (NC), si-NC, Ezrin-overexpression and si-Ezrin plasmids (Sangon Biotech, China) using Lipofectamine 2000 (Promega, USA).

### 2.5. CTC isolation

Isolation and enumeration of CTCs from peripheral blood samples were performed using the Veridex Cell Search® assay (Veridex, USA). The sample preservative tubes were centrifuged to collect the cell pellets. The supernatant was discarded, and the cell pellets were suspended by adding 5 mL of PBS. Blood samples (collected within 4 h) were filtered through a calibrated membrane with 8-μm-diameter pores (Millipore, Billerica, USA). To meet the need for filtration, we applied a filtration system consisting of a filtration tube containing the membrane (SurExam, Guangzhou, China), a manifold vacuum plate with valve settings (SurExam, Guangzhou, China), an E-Z 96 vacuum manifold (Omega, Norcross, USA), and a vacuum pump (Auto Science, Tianjin, China). Then, the pump valve was switched on to reach at least 0.08 MPa, and the manifold vacuum plate valve was then switched on to perform filtration. The circulating tumor cells were retained on the filter, and the blood cells went through the pores based on the fact that CTCs are larger than blood cells. Red blood cell lysis buffer (154 mM NH_4_Cl, 10 mM KHCO_3_ and 0.1 mM EDTA) was used to remove possible residual erythrocytes. The CTCs were fixed with 2% formaldehyde.

Briefly, 2 proprietary Cell Save tubes were used to hold the whole blood of patients. Nucleic acid fluorescent dyes (4ʹ,6-diamidino-2-phenylindole) conjugated with CK8, 18, 19 and lacking CD45 antibodies were added and incubated with the cells. After magnetic separation, the CTCs were immobilized onto the magnetic beads, and the CTCs were subjected to identification and quantification using Cell Tracks Analyzer II. Samples ≥ 5 CTCs/7.5 ml were defined as CTC positive; otherwise, they were defined as negative.

### 2.6 Tri-color RNA in situ hybridization (ISH)assay

The assay was performed in a 24-well plate (Corning, NY, USA), and the cells on the membrane were treated with protease before hybridization with capture probe specific for the EpCAM and CK8, the leukocyte biomarker CD45 and Ezrin (sequences shown below). The hybridization was performed at 42°C for 2 h, and then unbound probes were removed by washing 3 times with washing buffer (0.1xSSC). Then the signal amplification step was performed by incubating with 100 μl of preamplifier solution (30% horse serum, 1.5% sodium dodecylsulfate, 3 mM Tris-HCl (pH8.0) at 42°C for 20 minutes. The membranes were cooled, washed three times with wash buffer (0.1× SSC), and then incubated with 100 μl of amplifier solution. Three types of fluorescently labeled probes were conjugated with the membrane.

**Table:** 

Gene	Sequences (5ʹ-3ʹ)
Ezrin	TTGAACTGGAGGGGATTCTCTTCCTTCACTTGGAGGAAGAGCAG TAGATCTCATCGCTAACAAGAGCACGGCAGTCTCAGTGTTGTA GTCCCAAACTTGCTGAGGTACCCAGACTTGTGACTCTTTGAGG GATCAGCCGAGCATTATCTTTGAGCATCC
EpCAM	TGGTGCTCGTTGATGAGTCAAGCCAGCTTTGAGCAAATGA AAAGCCCATCATTGTTCTGGCTCTCATCGCAGTCAGGATCT CCTTGTCTGTTCTTCTGACCTCAGAGCAGGTTATTTCAG
Ck8	CGTACCTTGTCTATGAAGGAACTTGGTCTCCAGCATCTTGC CTAAGGTTGTTGATGTAGCCTGAGGAAGTTGATCTCGTCCA GATGTGTCCGAGATCTGGTGACCTCAGCAATGATGCTG
CD45	TCGCAATTCTTATGCGACTCTGTCATGGAGACAGTCATGTG TATTTCCAGCTTCAACTTCCCATCAATATAGCTGGCATTTTG TGCAGCAATGTATTTCCTACTTGAACCATCAGGCATC

### 2.7. Transwell assays

Transwell chambers were coated with Matrigel (BD Biosciences) with the 8-µm chamber holes in the membrane filter. Briefly, the cells transfected with NC or Ezrin-overexpression plasmids were resuspended in serum-free culture medium. These cells, with a density of 150,000 cells, were seeded into the upper chambers. RPMI‑1640 medium supplemented with 20% FBS, as a chemoattractant, was added to the lower chambers. After 24 h of incubation, cells in the upper chambers were removed, and the cells in the lower chambers were collected and fixed with 4% paraformaldehyde and finally stained with crystal violet (0.5%). The stained cells were counted from 5 random fields.

### 2.8. Statistical analysis

All data were processed using SPSS 18.0 and GraphPad Prism software. Data are shown as the mean ± SD of at least triplicate experiments. Student’s two-tailed *t*-test was performed to compare data between two groups, and one-way ANOVA was performed to compare data between 3 groups. *P < *0.05 was regarded to be statistically significant.

## 3. Results

### 3.1. Ezrin expression was upregulated in tissue and cell samples of human prostate cancer

**Figure 1.**
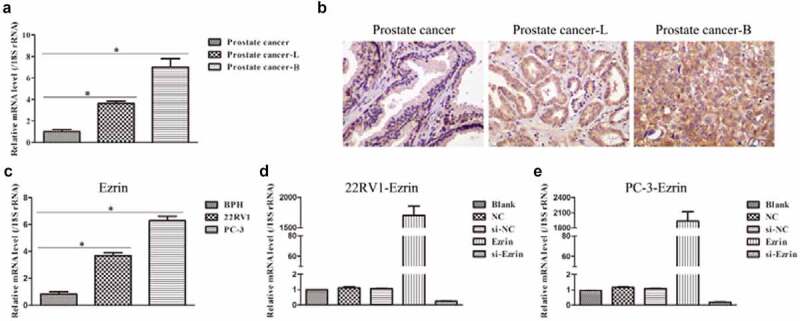
Expression of Ezrin in prostate cancer tissues and cell lines. (a) The Ezrin expression in different stages of PCa was determined by qRT-PCR. *P < 0.05. (b) IHC staining of Ezrin was performed on PCa tissues at different stages. (c) The relative expression of Ezrin in different PCa cell lines. *P < 0.05. (d) 22RV1 cells were transfected with different plasmids to evaluate the Ezrin expression with qRT-PCR. (e) Different plasmids were transfected to PC-3 cells to assess the Ezrin expression using qRT-PCR.

To investigate the role of Ezrin in human prostate cancer, the mRNA level of Ezrin from the collected tissue samples was first examined by qRT–PCR and IHC staining. The results showed that the mRNA expression of Ezrin was significantly increased in the prostate cancer-lymph node metastasis (PCa-L) and the prostate cancer-bone metastasis (PCa-B) groups compared with the prostate cancer group (Figure 1(a)). Moreover, the mRNA expression of Ezrin was highest in the prostate cancer-B group. Additionally, IHC staining showed 3 types of Ezrin expression: faint cytoplasmic staining in scattered cells indicated weak expression in the prostate cancer group, heterogeneous cytoplasmic staining in tumor cells indicated moderate expression in the prostate cancer-lymph node metastasis group, and dense cytoplasmic staining in all tumor cells indicated strong expression in the prostate cancer-bone metastasis group (Figure 1(b)). Subsequently, we adopted three human prostate cancer cell lines, 22RV1 and PC-3, to further detect Ezrin expression. The data showed that Ezrin expression in 22RV1 and PC-3 cells was notably higher than that in prostate BPH cells (Figure 1(c)). Previous studies have reported that 22RV1 cells have lymph node metastasis features, while PC-3 cells have bone metastasis features. Therefore, the expression of Erzin in tissue samples and cell samples was consistent. Finally, Ezrin-overexpression and si-Ezrin plasmids were transfected into 22RV1 and PC-3 cells. It was found that 22RV1 and PC-3 cells transfected with Ezrin-overexpression plasmid markedly upregulated Ezrin expression, but 22RV1 and PC-3 cells transfected with si-Ezrin plasmid markedly downregulated Ezrin expression (Figure 1(d,e)). Therefore, the above data suggested that elevated Ezrin expression might promote the development of human prostate cancer.

### 3.2. Ezrin promoted the metastasis ability of PCa cells

**Figure 2.**
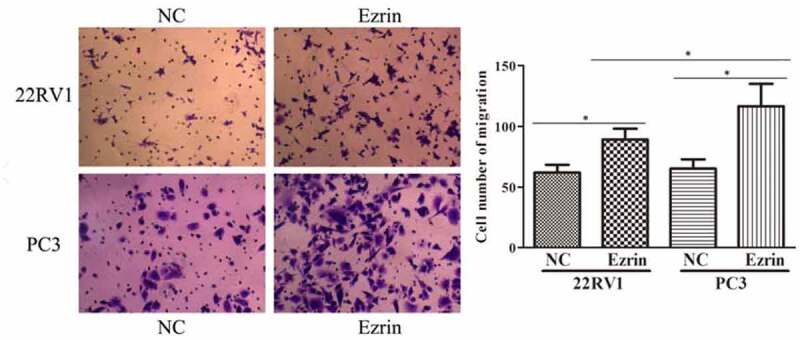
Transwell assays indicated marked cell migration in Ezrin-overexpression plasmid transfected 22RV1 and PC-3 cells, compared with the negative control (NC) cells. *P < 0.05.

**Figure 3.**
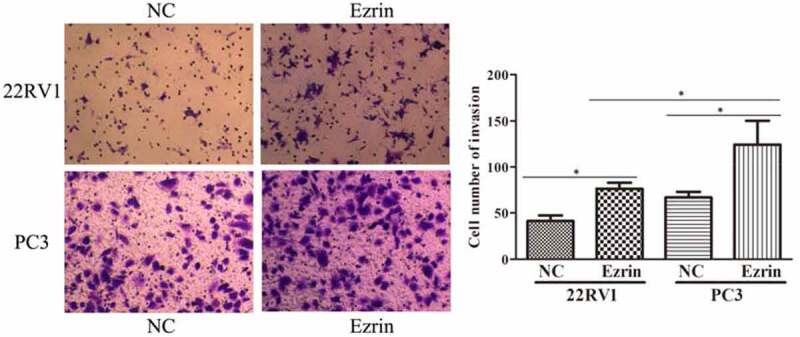
Transwell assays revealed marked cell invasion in Ezrin-overexpression plasmid transfected 22RV1 and PC-3 cells, compared with the negative control (NC)-tranfected cells. *P < 0.05.

Tumor metastasis, which is characterized by cell migration and invasion, is always an obstacle for advanced cancer treatment. In this study, to explore the effect of Ezrin on tumor metastasis in 22RV1 and PC-3 PCa cells, Transwell assays were performed. The migratory abilities of 22RV1 and PC-3 cells transfected with the Ezrin-overexpression plasmid were significantly enhanced compared to those of 22RV1 and PC-3 cells transfected with the NC plasmid (Figure 2). Furthermore, the invasive abilities of Ezrin-transfected 22RV1 and PC-3 cells were also apparently higher than those of 22RV1 and PC-3 cells transfected with NC plasmid (Figure 3). Additionally, the migration and invasion abilities of 22RV1 cells were markedly weaker than those of PC-3 cells. Hence, these data implied that strong Ezrin expression might correlate with the metastatic ability of PCa cells.

### 3.3. CTC counts and Ezrin expression in CTCs were upregulated in metastatic PCa

**Figure 4.**
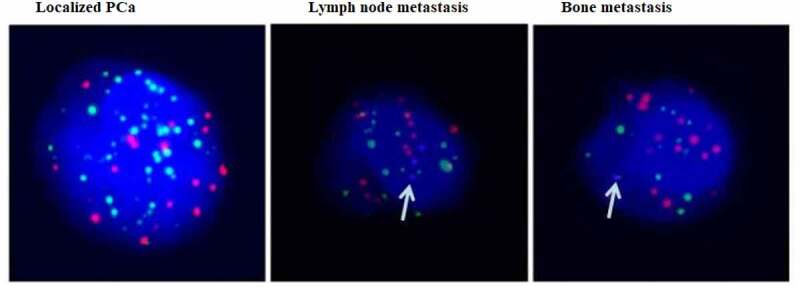
Ezrin expression (blue dots) in CTCs from different stage of PCa by using *in situ* hybridization.

PSA levels and the Gleason score (GS) are widely used to score worse oncological outcomes in PCa. We chose three different stages of PCa patients, namely, localized PCa, PCa with lymph node metastasis and PCa with bone metastasis. As shown in Table 1, there were very gradual increases in PSA levels and GS with tumor metastasis. Moreover, it was found that the mean number of CTCs was significantly increased in PCa with metastasis characteristics compared to localized PCa. Then, we identified Ezrin expression in CTCs of PCa. First, we verified the purity and characterized the isolated CTCs using the CanPatrol system. Fluorescence staining showed that the CTCs were consistent with previous reports, and the purity of CTCs was high. Moreover, Ezrin was markedly more abundant in CTCs of PCa with bone metastasis than in those of localized PCa (Figure 4). Thus, these findings indicated that Ezrin in CTCs of PCa might intimately correlate with the metastatic process.

### 3.4. The different CTC subsets in various stages of PCa

As illustrated in Table 2, epithelial CTCs were the main CTC subset in localized PCa, while mesenchymal CTCs and mixed cell type CTCs were relatively lower in localized PCa. However, in metastatic PCa, mesenchymal CTCs and mixed cell type CTCs were sharply elevated. Therefore, these data concluded that mesenchymal CTCs and mixed cell type CTCs might participate in metastatic PCa. It can be concluded that Ezrin is a potential prognostic marker for PCa diagnosis.

**Table 2.** CTC subtypes’ analysis on the 80 PCa patients

	Localized PCa	PCa with lymph node metastasis	PCa with bone metastasis	*P* values
Total of CTCs	2.2 (0 ~ 13)	5.6 (0 ~ 20)	10.8 (5 ~ 48)	*P* < 0.05
Epithelial CTCs	1.3 (0 ~ 6)	2.9 (0 ~ 10)	3.4 (1 ~ 20)	*P* < 0.05
Mesenchymal CTCs	0.4 (0 ~ 3)	1.3 (0 ~ 8)	5.6 (4 ~ 13)	*P* < 0.05
E/M mixed CTCs	0.5 (0 ~ 4)	1.5 (1 ~ 6)	2.8 (2 ~ 17)	*P* < 0.05
Ezrin expression	0.2 (0 ~ 2)	3.5 (2 ~ 8)	12 (6 ~ 27)	*P* < 0.01

### 3.5. The prognostic functions of Ezrin in CTCs of PCa

To further investigate the functions of Ezrin in CTCs of PCa, we selected 53 PCa patients who underwent radical prostatectomy (Table 3). After surgery, PCa patients with Ezrin-positive expression presented distinctly higher PSA levels, more serious T status and higher GS than PCa patients with Ezrin-negative expression.

**Table 3.** Characteristics of 53 cases of radical prostatectomy and analysis on the expression of Ezrin in CTCs of these cases

	Total information	CTC-Ezrin (+)	CTC-Ezrin (-)
Cases (N)	53	45	8
Age (Years)	68 (50 ~ 84)	69 (52 ~ 83)	67 (50 ~ 84)
PSA (ng/ml)≤10	13	9	4
10< PSA(ng/ml)≤20	27	25	2
PSA(ng/ml)>20	13	11	2
T status after surgery<T2c	15	11	4
T status after surgery≥T2c	38	34	4
GS after surgery<7	8	6	2
GS after surgery = 7	28	24	4
GS after surgery>7	17	15	2
Edge +	2	2	0
Lymph node+	11	10	1

## 4. Discussion

Metastasis is a multistep process, in which the tumor cells first disseminate from primary sites and subsequently arrive in a distant organ to form a new tumor mass. This process is the predominant cause of mortality from malignant cancers, including PCa [22,23]. Therefore, exploring the potential mechanism of tumor metastasis in PCa could significantly decrease the mortality rate of PCa. Ezrin, the first ERM protein identified, is involved in cell adhesion, survival, proliferation and migration to the extracellular matrix, which suggests that Ezrin might be associated with the metastatic process of tumors. For instance, Feng et al. verified that Ezrin expression was notably associated with clinical progression indicators in gastric and colorectal cancers [24]. Nevertheless, the molecular mechanisms of Ezrin in PCa metastasis have still not been reported. In this study, it was discovered that Ezrin expression in PCa samples with lymph node and bone metastasis characteristics was significantly higher than that in localized PCa samples. Moreover, the expression of Ezrin in PCa cells with lymph node and bone metastasis features was also apparently higher than that in PCa cells with nonmetastatic features. These results suggested that Ezrin might play a crucial role in the development of PCa. Migration and invasion are the main biological behaviors during the metastatic process of PCa. Subsequently, we further examined the roles of Ezrin in PCa mobility. The results showed that forced Ezrin expression could markedly upregulate the migration and invasion characteristics of PCa cells; therefore, these results indicated that Ezrin might be a key biomarker in PCa metastasis.

Previous studies have reported that CTCs are already regarded as a promising marker with prognostic and predictive value for potential clinical outcome and therapy response in urological cancers, such as PCa, kidney cancer and bladder cancer [25–27]. In this study, it was revealed that the higher the CTC count was, the more serious the stage of PCa, which implied that there might be a positive relationship between the CTC count and the metastasis degree of PCa. Moreover, the Ezrin expression of CTCs in metastatic PCa was notably higher than that in localized PCa, which illustrated that Ezrin expression in CTCs might be an indicator during PCa progression. Additionally, CTC subtype analysis further showed that the predominant cell subtypes in localized PCa and metastatic PCa were epithelial CTCs and mesenchymal/mixed cell type CTCs, respectively. Emerging evidence has demonstrated that EMT in the development of cancers could enable cancer dissemination and metastatic spread [28,29]. Thus, our results hinted that mesenchymal and mixed cell type CTCs represented the metastasis process of PCa. Finally, to further verify the influence of positive or negative Ezrin expression in CTCs of PCa, we evaluated radical prostatectomy prognosis in PCa patients. The data revealed that PCa patients with positive Ezrin expression might be susceptible to reoccurrence after radical prostatectomy; therefore, the results may be implicated in androgen-independent recurrence, which leads to CRPC. To further investigate the role of Ezrin in prostate cancer, the upstream and downstream networks should be analyzed. More clinical research and data collection should also be performed in the future to prove the diagnostic value of Ezrin in prostate cancer.

## 5. Limitations

In this study, only the biological function of Ezrin was explored; the molecular network would be more important for us to investigate. The survival rate in prostate cancer patients with high expression or low expression of Ezrin was not followed.

## 6. Conclusion

In this study, we found that both CTCs and Ezrin were closely related to PCa metastasis, which were collectively responsible for predicting the metastatic process of PCa. Therefore, these results indicated that Ezrin expression in CTCs may offer novel insights into the prognosis and management of PCa.
